# Microendoscopy-assisted extraforaminal lumbar interbody fusion for treating single-level spondylodesis

**DOI:** 10.1186/s13018-021-02313-9

**Published:** 2021-03-02

**Authors:** Motohide Shibayama, Guang Hua Li, Li Guo Zhu, Zenya Ito, Fujio Ito

**Affiliations:** 1 Department of Orthopedic Surgery, Aichi Spine Hospital, 31-1 Kamiike Goroumaru, Inuyama City, Aichi Japan; 2 Department of Orthopedic Surgery, Wang Jing Hospital of CACMS, Beijing, China

**Keywords:** Extraforaminal lumbar interbody fusion, Microendoscope, Minimally invasive, Lumbar spondylodesis

## Abstract

**Background:**

Lumbar interbody fusion is a standard technique for treating degenerative lumbar disorders involving instability. Due to its invasiveness, a minimally invasive technique, extraforaminal lumbar interbody fusion (ELIF), was introduced. On surgically approaching posterolaterally, the posterior muscles and spinal canal are barely invaded. Despite its theoretical advantage, ELIF is technically demanding and has not been popularised. Therefore, we developed a microendoscopy-assisted ELIF (mELIF) technique which was designed to be safe and less invasive. Here, we aimed to report on the surgical technique and clinical results.

**Methods:**

Using a posterolateral approach similar to that of lateral disc herniation surgery, a tubular retractor, 16 or 18 mm in diameter, was placed at the lateral aspect of the facet joint. The facet joint was partially excised, and the disc space was cleaned. A cage and local bone graft were inserted into the disc space. All disc-related procedures were performed under microendoscopy. The spinal canal was not invaded. Bilateral percutaneous screw-rod constructs were inserted and fixed.

**Results:**

Fifty-five patients underwent the procedure. The Oswestry Disability Index and visual analogue scale scores greatly improved. Over 90% of the patients obtained excellent or good results based on Macnab’s criteria. There were neither major adverse clinical effects nor the need for additional surgery.

**Conclusions:**

mELIF is minimally invasive because the spinal canal and posterior muscles are barely invaded. It produces good clinical results with fewer complications. This technique can be applied in most single-level spondylodesis cases, including those involving L5/S1 disorders.

## Background

Posterior interbody fusion is a standard technique for treating degenerative lumbar disorders. Posterior lumbar interbody fusion (PLIF) was developed first [1], followed by transforaminal interbody fusion (TLIF) [2]. These techniques have produced stable outcomes but are relatively invasive because both the posterior muscles and spinal canal are surgically invaded. Therefore, a minimally invasive technique, extraforaminal lumbar interbody fusion (ELIF), was introduced [3]. In this technique, the approach is from the posterolateral direction, and the disc is manipulated through Kambin’s safety triangle [4]. This damages the posterior muscles to a small extent, and it does not involve the surgical invasion of the spinal canal. However, despite its theoretical superiority, ELIF has not been popularised because it is technically difficult to perform.

**Fig. 1:**
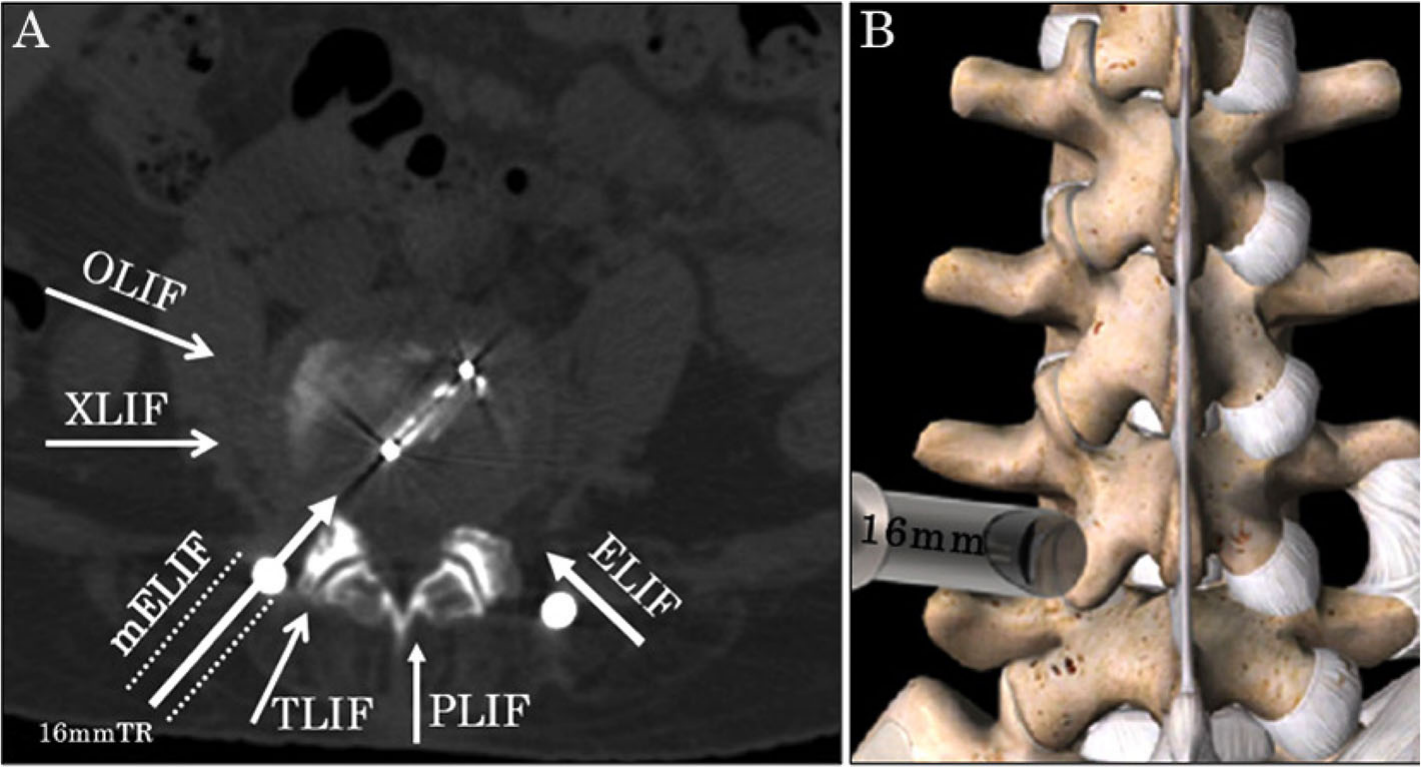
Schema of the approach. **a** Schema on a CT axial image. The approach for mELIF is more lateral than that of TLIF. The spinal canal is not surgically invaded. **b** Drawing from the posterior direction. A TR is placed on the lateral edge of the facet joint. Abbreviations: CT, computed tomography; mELIF, microendoscopy-assisted extraforaminal lumbar interbody fusion; TLIF, transforaminal lumbar interbody fusion; TR, tubular retractor; OLIF, oblique lumbar interbody fusion; XLIF, extreme lumbar interbody fusion; PLIF, posterior lumbar interbody fusion

Thus, we developed a unique single-level microendoscopy-assisted ELIF technique (mELIF). In this technique, the approach is more lateral than that of TLIF (Fig. 1a, b). In most of the procedure can be performed with a tubular retractor (TR) under endoscopic visual assistance. We considered that this technique could yield good results and reduce approach-related complications. Therefore, in this report, we aimed to describe the surgical technique and its clinical results.

## Patients and methods

A total of 55 patients (17 men and 38 women; mean age, 62.7 years; range, 43–79 years) underwent the mELIF procedure between 2015 and 2020. The index diagnoses were degenerative spondylolisthesis (*n* = 33), isthmic spondylolisthesis (*n* = 9), foraminal stenosis (*n* = 10), and others. Thirty-three patients had Meyerding grade II spondylolisthesis, and nine had grade I. Seven patients had previous canal decompression surgery. Thirty-six patients underwent surgery at L4/5. The remainder underwent surgery at L5/S1 (*n* = 16) and L3/4 (*n* = 3). To evaluate the levels of lumbar and radicular pain, changes in pain density were recorded using a visual analogue scale (VAS) with scores ranging from 0 to 10 (0: no pain and 10: the most severe pain). Physical spinal function was evaluated using the Oswestry Disability Index (ODI). The final outcome was evaluated using Macnab’s criteria. All the patients, apart from two who were lost to follow-up, were followed up for at least half a year. Demographic data are shown in Table 1. The surgical procedure is described below.

**Table 1:** Patient characteristics

Characteristic	Value
Mean age (years)	62.7 ± 11.6
Number	55
Male:Female	17:38
Two patients lost to follow up	
Follow-up period in months	6-50
Mean follow-up period in months	24
Level treated	
L4/5	36
L5/S1	16
L3/4	3
Diagnosis	
Degenerative spondylolisthesis	33
Isthmic lumbar spondylolisthesis	9
Grade II slip 33/42 (78.6%)	
Foraminal stenosis	9
Discogenic pain	2
Lateral disc herniation plus spinal stenosis	2

### Surgical procedure

**Fig. 2:**
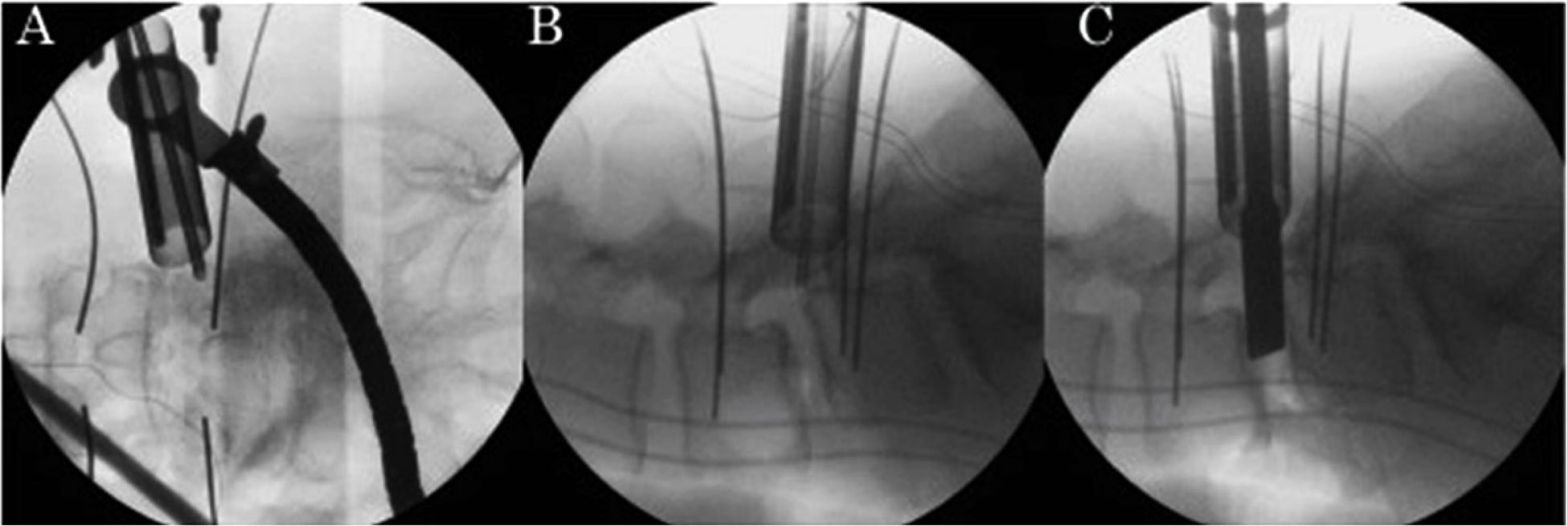
Fluoroscopic images during surgery. **a** A tubular retractor with a microendoscope installed posterolaterally on the anteroposterior view. Four percutaneous pedicle screw guide wires were already placed in the pedicles. **b** Approach to the disc on the lateral view. **c** The disc space was cleaned and prepared for cage insertion on the lateral view

mELIF at L4/5 with right-sided approach is described. Surgery was performed under general anaesthesia with the patient in the prone position. At approximately 5 cm from the midline, bilateral longitudinal skin incisions that were approximately 4 cm in length were made. Under fluoroscopic guidance, four guide wires were inserted bilaterally into the L4 and L5 pedicles for percutaneous pedicle screw placement. In the middle of the right-sided skin incision, approximately 2 cm of the fascia was cut. A TR with a diameter of 16 mm was docked on the lateral edge of the facet joint after sequential dilation (Fig. 2a). A microendoscope (Medtronic Sofamor Danek, Memphis, TN, USA) was then installed. Under the guidance of microendoscopic illumination and vision, the lateral aspect of the right L4/5 facet joint was excised with a chisel and preserved as graft bone. A high-speed burr was used for the final meticulous bone resection. The intertransverse process ligament was removed, and the right L4 exiting nerve root and disc were identified (Fig. 2b). The root was protected, and a rotational expander followed by a ring curette were inserted into the disc. The disc space was subsequently cleaned (Fig. 2c).

**Fig. 3:**
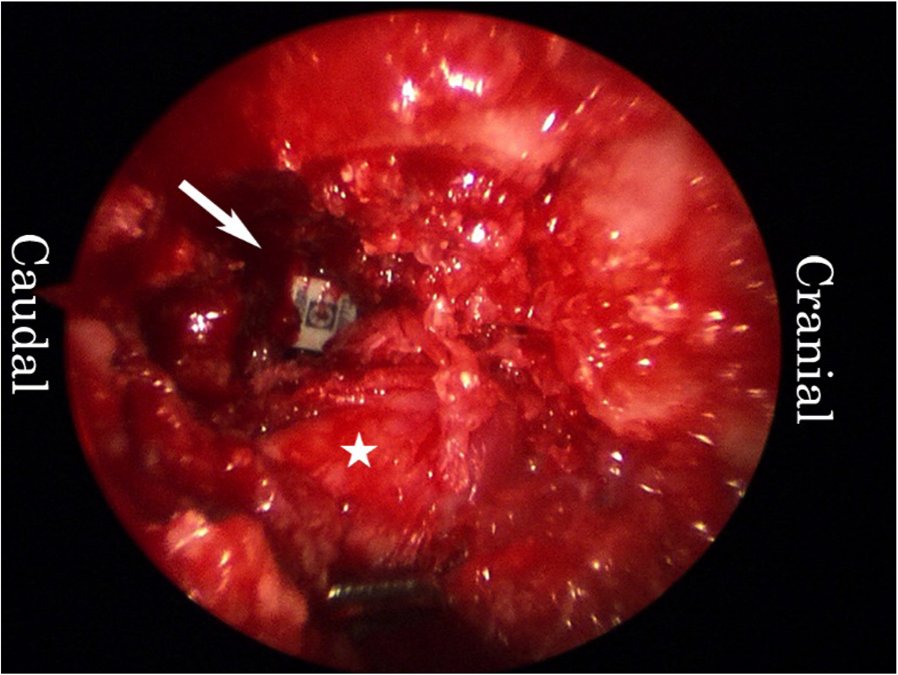
Endoscopic image. An interbody cage was inserted into the disc space. Arrow head: cage. Asterisk: right L5 exiting nerve root

Under fluoroscopic guidance, a mixture of bone graft and β-tricalcium phosphate was inserted into the disc space through the TR, followed by the insertion of a bullet-type cage (Fig. 3). After saline irrigation, the TR was removed. Bilateral pedicle screws and rod constructs were placed. A suction drain was placed only on the right side of the wound, and the wounds were closed.

### Statistical analysis

The Student *t* test was used for the analysis of the differences in the VAS score and the Mann-Whitney *U* test for the differences in the ODI. Results were considered statistically significant for a *P* value of < 0.05 in all the statistical test methods.

## Results

The average operative time and estimated blood loss were 160.6 ± 25.5 min and 70.5 ± 19.4 ml, respectively. The mean preoperative VAS score for lumbar pain was 5.9 ± 2.45, and the mean VAS score for radicular pain was 6.2 ± 2.42. The postoperative lumbar and radicular VAS scores improved to 1.6 ± 1.66 and 1.5 ± 1.69, respectively. The parameters used to assess lumbar and radicular pain relief (VAS score) improved significantly (*P* < 0.05). Spinal function evaluated by the ODI (%) measured 20.98 ± 5.04 preoperatively. This index improved to 8.85 ± 5.26 at 6 months and to 6.39 ± 4.64 at 1 year postoperatively (*P* < 0.05). Based on Macnab’s criteria, over 90% of the patients obtained excellent (*n* = 42) or good (*n* = 7) results whilst the rest exhibited fair (*n* = 3) or poor (*n* = 1) results. Regarding complications, asymptomatic cage migration was observed in three cases. There were no subsequent surgical procedures performed, and no other complications were observed (Table 2).

**Table 2:** Clinical results

	Preoperative	Postoperative	Significance
Visual analogue score			
Lumbar pain	5.9 ± 2.45	1.6 ± 1.66	*P = 0.0036
Radicular pain	6.2 ± 2.42	1.5 ± 1.69	*P = 0.0137
Oswestry Disability Index	Preoperative	Postoperative (6 mo)	Postoperative (12 mo)
	20.98 ± 5.04	10.46 ± 4.67	7.89 ± 4.46
Macnab criteria			
Excellent	42 (79%)		
Good	7 (13%)		
Fair	3 (5%)		
Poor	1 (2%)		
Mean estimated blood loss in ml	70.5 ± 19.4		
Mean operative time in minutes	160.6 ± 25.5		
Complications			
Cage migration without symptoms	3		
Dural tearing, hematoma, infection	0		
Internal organ injury, existing nerve root injury	0		

### Illustrative case

**Fig. 4:**
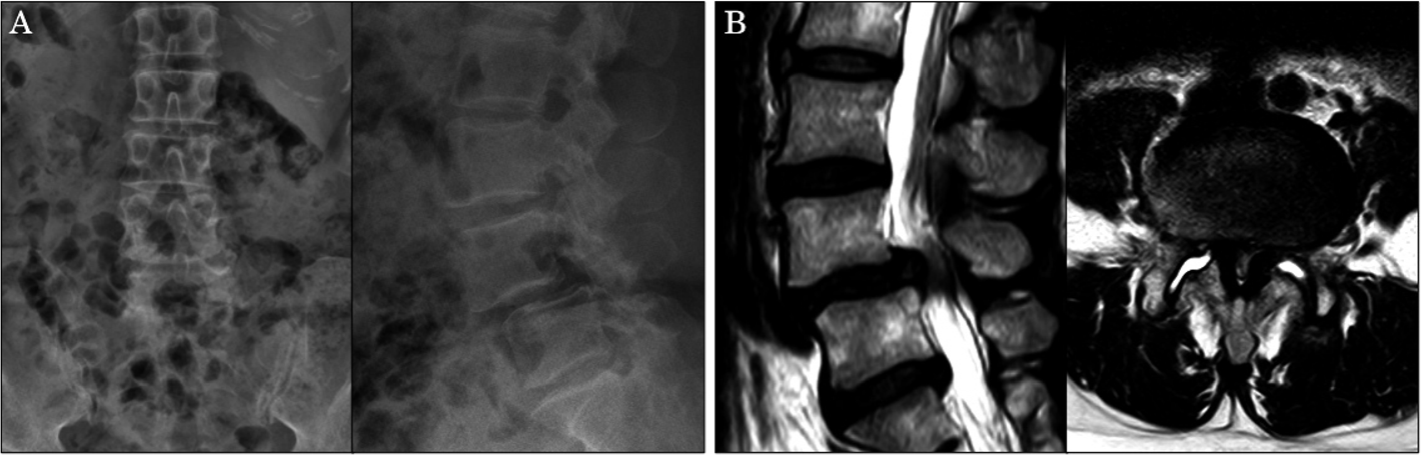
Images at presentation. **a** Anteroposterior and lateral X-ray films. Note grade II spondylolisthesis at L4/5. **b** Sagittal and axial magnetic resonance images. The spinal canal is severely compressed at L4/5

A 61-year-old woman presented at our hospital with a complaint of lower back pain and bilateral sciatica, which had lasted for 2 years. Imaging studies revealed grade II degenerative spondylolisthesis and severe spinal stenosis at L4/5 (Fig. 4a, b). The VAS scores were 7.0 for lumbar pain and 8.0 for radicular pain. The ODI was 40%.

### Postsurgical course

**Fig. 5:**
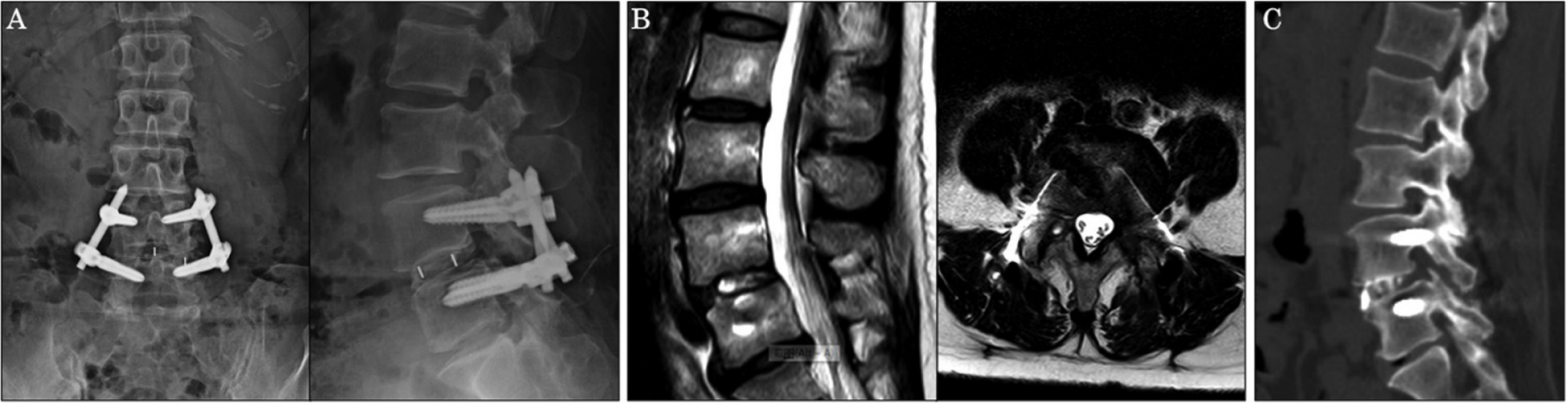
Images at 1-year post-surgery. **a** Anteroposterior and lateral X-ray films. Spondylolisthesis was corrected, and the hardware was in place. **b** Sagittal and axial magnetic resonance images. The spinal canal was decompressed without direct surgical decompression. **c** Computed tomography image showing the bone fused around the cage

The operative time was 206 min, and the amount of blood loss was 50 ml. The patient’s symptoms were greatly alleviated after surgery. During the final follow-up conducted 2 years after surgery, the VAS scores were 1.1 for lumbar pain and 0 for radicular pain. The ODI improved to 6% at 2 months and to 4% at 2 years post-surgery. The result was excellent based on Macnab’s criteria. One year after surgery, radiography demonstrated that a good correction of spondylolisthesis was maintained (Fig. 5a). Magnetic resonance imaging revealed that the spinal canal was decompressed without direct decompression (Fig. 5b), and computed tomography also revealed bone fused around the cage (Fig. 5c).

## Discussion

Lumbar interbody fusion is a standard technique for treating degenerative lumbar disorders. In 1952, the PLIF procedure was introduced by Cloward as a posterior type of lumbar interbody fusion technique [1]. The TLIF procedure was developed as an alternative to PLIF [2]. Later, numerous advances in posterior fusion techniques were introduced for clinical use [5–8]. The PLIF and TLIF techniques yield stable results. However, the procedures are relatively invasive as posterior interbody fusion techniques involve the need to manipulate the spinal canal, as a result of which dural tears or nerve injury can occur occasionally.

ELIF, a less invasive interbody fusion technique, was introduced by Phillips and Cunnigham [3] based on Wiltse’s approach [9] in 2002. The approach in ELIF is from the posterolateral direction which is more lateral compared to those in PLIF or TLIF (Fig. 1a). Interbody fusion is performed through Kambin’s safety triangle [4] without the need for surgically invading the spinal canal.

In theory, ELIF has many advantages: minimal invasiveness, no canal invasion, and easy revision surgery after the previous decompression of the canal. However, ELIF has not been popular. In our opinion, the reason for this is that ELIF is considered technically demanding because the surgical field is deep, and the illumination is poor. In addition, surgeons were concerned about damaging the dorsal root ganglion of the exiting nerve root. Therefore, Baek et al. [10] recommended a wide dissection and meticulous manoeuvre in ELIF.

Based on the same concept that does not involve the surgical invasion of the spinal canal, lumbar lateral interbody fusion (LLIF) was introduced [11–14]. LLIF employs a lateral approach, and neither the spinal canal nor the posterior lumbar muscles are surgically invaded. This technique demonstrates that ‘indirect neural decompression without direct decompression of the spinal canal’ can result in good clinical recovery. LLIF procedures, including oblique lateral interbody fusion (OLIF) [11, 12] and extreme lateral interbody fusion (XLIF) [13, 14], have become increasingly popular. LLIF has been demonstrated to produce good results in a minimally invasive manner, but the rates of approach-related adverse effects, some of which are very severe, have been reported to be relatively high [15–17]. In XLIF which involves a lateral retroperitoneal transpsoas approach, lumbar plexopathy, bowel injuries, ureteral injuries and vascular injuries have been reported [15, 16]. OLIF involves the use of a peritoneal approach, and complications, such as lumbar plexopathy, peritoneal lacerations and ureteral injuries, have been reported [16, 17].

mELIF is a unique lumbar interbody fusion technique that entails the use of the ELIF approach under spinal microendoscopy. The skin incision is made 6 to 10 cm from the midline in ELIF [10, 18] and 4 to 6 cm from the midline in mELIF. As stated by Baek et al. [10], a wide dissection is needed in ELIF, and in contrast, minimal invasiveness can be maintained in mELIF by using a 16 or 18mm TR. In mELIF, the approach angle is approximately 45 degrees from the posterolateral direction, which is away from the internal organs and psoas muscles. The procedure is performed under bright and clear microendoscopic vision. Therefore, we believe that mELIF is a safe surgical technique.

Microendoscopic discectomy was invented by Foley and Smith in 1997 [19]. In this system, surgery is performed using a TR with a diameter of 16 or 18mm and a microendoscope. The indications for this technique have been expanded to include lumbar spinal stenosis [20], lateral disc herniation [21] and extraforaminal stenosis [22, 23]. We employed the approach used in lateral disc herniation or extraforaminal stenosis in mELIF.

Another endoscopic ELIF technique performed through Kambin’s safety triangle was also reported [24, 25]. In this technique, the approaches are relatively distant from the midline and are close to those involved in the original ELIF procedure. They used full endoscopy (FES) of which diameter is around 8 mm. The advantage of mELIF is that manipulations such as removing bone, securing nerve root and inserting a cage into the disc are clearly seen in 16 mm TR. These procedures are difficult to see directly in fusion surgeries using FES. Therefore, we believe that mELIF can be performed in a safer manner. Another benefit of mELIF is that bone resection is easier. Therefore, complicated cases such as with L5/S1 disorder or with grade II spondylolisthesis can be good candidates for mELIF.

We performed a single-level mELIF procedure in 55 patients, and the results were very favourable. The results in over 90% of the cases were excellent or good. We are certain that indirect decompression is also valid in mELIF as well as in other LLIF techniques. There were neither major clinical complications nor the need to perform revision surgical procedures in this study. Three patients developed asymptomatic cage migration in the disc space. Bone union was obtained in 90% of the cases at 2 years postoperatively. The rate was comparable to that of TLIF studies [5–8].

The main index diagnoses in this study were degenerative spondylolisthesis (*n* = 33), isthmic spondylolisthesis (*n* = 9) and foraminal stenosis (*n* = 10). We believe that mELIF can be used for treating most of the single-level lumbar degenerative disorders, including Meyerding grade II spondylolisthesis. One big advantage of mELIF is that with a little additional effort, it can be applied for treating L5/S1 disorders by removing the sacral ala. LLIF techniques, including OLIF and XLIF, are usually not suitable for treating L5/S1 issues for anatomical reasons. There are other advantages of mELIF. The local bone can be kept and used as an autograft. In mELIF performed at L4/5 and L5/S1, if the local graft bone obtained is not enough, additional bone can be easily obtained from the nearby ilium, and because the skin incision is close to the iliac crest, another incision is not necessary. There is no need for changing the patient’s position during mELIF, which is usually needed during XLIF and OLIF procedures. There is no need for changing the patient’s position during mELIF, which is usually needed during XLIF and OLIF procedures. We summarise advantage and disadvantage of various fusion techniques in Table 3.

**Table 3:** Advantage and disadvantage of various interbody fusion techniques

	mELIF	PLIF	TLIF	OLIF	XLIF	ELIF
Minimally invasiveness	◎	X	○	◎	◎	○
Technical easiness	○	△	○	○	○	X
Corrective strength	○	◎	○	◎	◎	○
Bone union	○	◎	○	◎	◎	○
Safety	◎	△	○	△	△	△
L5/S1	◎	◎	◎	X	X	△
Position change	◎	◎	◎	X	X	◎

Classification based on our experience and opinions. ◎: Excellent, ○: Good, △: Fair, X: Poor

**Fig. 6:**
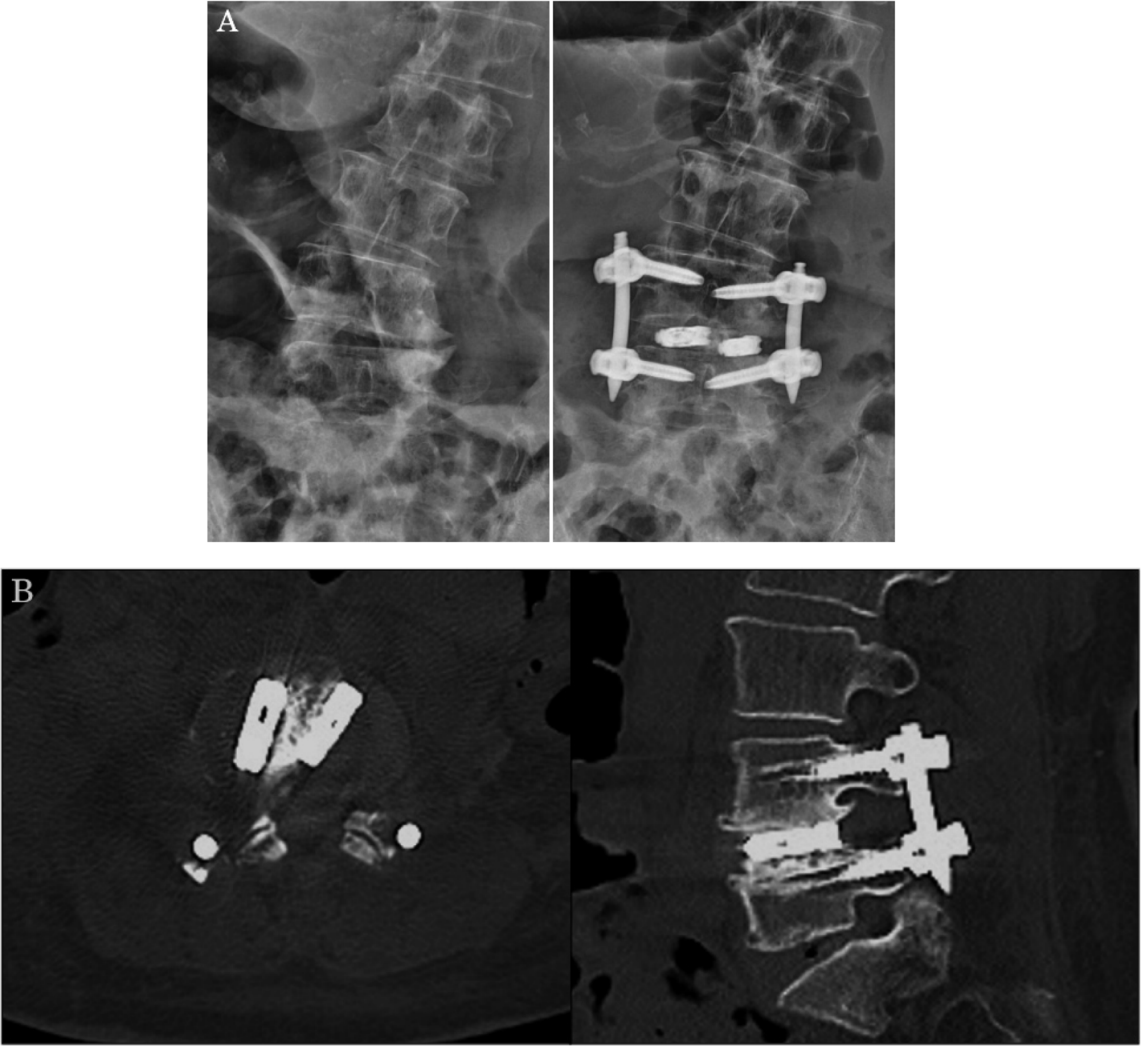
Insertion of two cages. **a** Anteroposterior X-ray films before and after surgery. **b** Computed tomography sagittal and axial images

There were several shortcomings associated with mELIF in this study. This procedure was only applied to treat single-level disorders. Multiple-level mELIF can be performed, but we do not have the experience yet. The operative time was also slightly prolonged, but after our initial experience, we are certain that this can be shortened. We used a single bullet-type cage in most of the cases, and the correction was not as strong as that achieved in the XLIF and OLIF procedures, in which a bigger cage is used. However, recently, we managed to insert two cages in several cases (Fig. 6a, b). In this study, bone union was achieved in 78% of the cases at 1-year post-surgery and in 90% at 2 years. This number is comparable to that of TLIF studies [5–8].

In addition, there are other limitations in this investigation. There was no control group in this study. The number of cases was relatively low. The follow-up period was relatively short. Further follow-up is necessary to assess long-term outcomes. However, our preliminary results appear promising. In conclusion, mELIF is safe and produces stable results in a minimally invasive manner, and therefore, can be used as an alternative to other more invasive lumbar interbody fusion techniques for the treatment of patients with single-level spondylodesis, including those with L5/S1 disorders.

## Data Availability

Informed consent for publication availability of data and materials was obtained from all participants.

## Abbreviations

ELIF: Extraforaminal lumbar interbody fusion
mELIF: Microendoscopy-assisted extraforaminal lumbar interbody fusion
PLIF: Posterior lumbar interbody fusion
TLIF: Transforaminal interbody fusion
TR: Tubular retractor
VAS: Visual analogue scale
ODI: Oswestry Disability Index
LLIF: Lumbar lateral interbody fusion
OLIF: Oblique lateral interbody fusion
XLIF: Extreme lateral interbody fusion

## References

[1] Cloward RB (1953). The treatment of ruptured intervertebral discs by vertebral body fusion. I. Indications, operative technique, after care. Am J Surg.

[2] Harms JG, Jeszenszky D (1998). The unilateral transforaminal approach for posterior lumbar interbody fusion. Orthop Traumatol.

[3] Phillips FM, Cunnigham B (2002). Intertransverse lumbar interbody fusion. Spine..

[4] Kambin P, Brager MD (1987). Percutaneous posterolateral discectomy: anatomy and mechanism. Clin Orthop Relat Res.

[5] Foley KT, Langson TH, Schwender JD (2003). Minimally invasive lumbar spine fusion. Spine..

[6] Isaacs RE, Podichetty VK, Santiago P (2005). Minimally invasive micro-endoscopy-assisted transforaminal lumbar interbody fusion with instrumentation. J Neurosurg Spine.

[7] Mummaneni PV (2005). The mini-open transforaminal lumbar interbody fusion. Oper Neurosurg.

[8] Foley KT, Gupta SK (2002). Percutaneous pedicle screw fixation of the lumbar spine: preliminary clinical results. J Neurosurg.

[9] Wiltse LL, Spencer CW (1988). New uses and refinements of the paraspinal approach to the lumbar spine. Spine..

[10] Baek OK, Lee SH (2009). Extraforaminal lumbar interbody fusion for the treatment of isthmic spondylolisthesis. J Spinal Disord Tech.

[11] Mayer HM (1997). A new microsurgical technique for minimally invasive anterior lumbar interbody fusion. Spine..

[12] Mehren C, Mayer HM, Zandanell C, Siepe CJ, Korge A (2016). The oblique anterolateral approach to the lumbar spine provides access to the lumbar spine with few early complications. Clin Orthop Relat Res.

[13] Ozgur BM, Aryan HE, Pimenta L, Taylor WR (2006). Extreme lateral interbody fusion (XLIF): a novel surgical technique for anterior lumbar interbody fusion. Spine..

[14] Mundis GM, Akbarnia BA, Phillips FM (2010). Adult deformity correction through minimally invasive lateral approach techniques. Spine..

[15] Rodgers WB, Gerber EJ, Patterson J (2011). Intraoperative and early postoperative complications in extreme lateral interbody fusion. An analysis of 600 cases. Spine..

[16] Uribe JS, Deukmedjian AR (2015). Visceral, vascular, and wound complications following over 13,000 lateral interbody fusions: a survey and literature review. Eur Spine J.

[17] Fujibayashi S, Kawakami N, Asazuma T (2017). Complications associated with lateral interbody fusion: nationwide survey of 2998 cases during the first 2 years of its use in Japan. Spine..

[18] Recoules-Arche D, Druschel C, Fayada P, Vinikoff L, Disch AC (2016). Unilateral extraforaminal lumbar interbody fusion (ELIF): surgical technique and clinical outcome in 107 patients. Clin Spine Surg.

[19] Foley KT, Smith MM (1997). Microendoscopic discectomy. Tech Neurosurg.

[20] Khoo LT, Fessler RG (2002). Microendoscopic decompressive laminotomy for the treatment of lumbar stenosis. Neurosurgery..

[21] Foley KT, Smith MM, Rampersaud YR (1999). Microendoscopic approach to far lateral lumbar disc herniation. Neurosurg Focus.

[22] O'Toole JE, Eichholz KM, Fessler RG (2007). Minimally invasive far lateral micro-endoscopic discectomy for extraforaminal disc herniation at the lumbosacral junction: cadaveric dissection and technical case report. Spine J.

[23] Matsumoto M, Chiba K, Ishii K, Watanabe K, Nakamura M, Toyama Y (2006). Microendoscopic partial resection of the sacral ala to relieve extraforaminal entrapment of the L-5 spinal nerve at the lumbosacral tunnel. Technical note. J Neurosurg Spine.

[24] Nakamura S, Taguchi M (2017). Full percutaneous lumbar interbody fusion: technical note. J Neurol Surg A Cent Eur Neurosurg.

[25] Youn MS, Shin JK, Goh TS, Lee JS (2018). Full endoscopic lumbar interbody fusion (FELIF): technical note. Eur Spine J.

